# Supplementary material for: Estimated Treatment Effects of Tight Glycaemic Targets in Mild Gestational Diabetes Mellitus: A Multiple Cut-Off Regression Discontinuity Study Design
Source: Int J Environ Res Public Health. 2020 Oct 22;17(21):7725. doi: 10.3390/ijerph17217725 (PMC7660094; doi:10.3390/ijerph17217725)
Supplement: Supplementary file 1 [file ijerph-17-07725-s001.pdf]

Supplementary table: Incidence of Large for Gestational Age (Birth weight >90th percentile) Newborns in Women Diagnosed with Gestational Diabetes Mellitus

HAPO Glucose Composite Score	Events (n)	Number of patients (N)
7	0	1
8	0	4
9	0	9
10	0	8
11	4	31
12	1	20
13	1	22
14	0	20
15	0	16
16	0	9
17	0	7
18	0	4
19	1	1
